# Supplementary material for: Long-Term Exposure to Ozone and Fine Particulate Matter and Risk of Premature Coronary Artery Disease: Results from Genetics of Atherosclerotic Disease Mexican Study
Source: Biology (Basel). 2022 Jul 27;11(8):1122. doi: 10.3390/biology11081122 (PMC9332787; doi:10.3390/biology11081122)
Supplement: Supplementary file 1 [file biology-11-01122-s001.zip › biology-1775168-supplementary.pdf]

Supplemental Table S1. Sensitivity analyses with further adjustment for ozone or $PM_{2.5}$ in the matching time window.

	1-year		2-year		3-year		4-year		5-year	
	**OR (95% CI)**	**p-Value**	**OR (95% CI)**	**p-Value**	**OR (95% CI)**	**p-Value**	**OR (95% CI)**	**p-Value**	**OR (95% CI)**	**p-Value**
Ozone	1.20 (1.11 – 1.31)	**<0.0001**	1.19 (1.05 – 1.35)	**0.006**	1.16 (1.02 – 1.33)	**0.02**	0.99 (0.88 – 1.11)	0.89	1.07 (0.97 – 1.19)	0.18
$PM_{2.5}$	0.46 (0.29 – 0.72)	**0.0007**	0.83 (0.44 – 1.57)	0.56	1.14 (0.54 – 2.43)	0.73	1.68 (0.78 – 3.61)	0.18	1.97 (0.89 – 4.36)	0.09
Relative humidity	0.98 (0.94 – 1.03)	0.45	1.09 (1.01 – 1.19)	**0.02**	0.96 (0.88 – 1.05)	0.39	1.05 (0.95 – 1.17)	0.30	1.01 (0.90 – 1.13)	0.85
Temperature	1.49 (1.12 – 1.98)	**0.006**	1.77 (1.18 – 2.67)	**0.006**	1.20 (0.73 – 2.00)	0.47	0.68 (0.40 – 1.17)	0.17	0.91 (0.53 – 1.55)	0.72
Wind velocity	0.62 (0.28 – 1.34)	0.22	0.60 (0.19 – 1.83)	0.37	0.44 (0.13 – 1.50)	0.19	0.27 (0.08 – 0.92)	**0.04**	0.57 (0.16 – 1.99)	0.38
BMI	0.95 (0.92 – 0.99)	**0.007**	0.96 (0.92 – 0.99)	**0.01**	0.96 (0.93 – 0.99)	**0.02**	0.96 (0.93 – 0.99)	**0.02**	0.96 (0.93 – 0.99)	**0.02**
Sex	10.30 (7.12 – 15.11)	**<0.0001**	10.47 (7.27 – 15.30)	**<0.0001**	10.08 (7.01 – 14.71)	**<0.0001**	10.08 (7.01 – 14.70)	**<0.0001**	10.13 (7.00 – 14.66)	**<0.0001**
Age	1.01 (0.99 – 1.03)	0.34	1.01 (0.99 – 1.03)	0.14	1.01 (0.99 – 1.03)	0.18	1.01 (1.00 – 1.03)	0.11	1.01 (0.99 – 1.03)	0.14
Junior high school	0.45 (0.31 – 0.65)	**<0.0001**	0.44 (0.30 – 0.64)	**<0.0001**	0.46 (0.32 – 0.66)	**<0.0001**	0.46 (0.32 – 0.66)	**<0.0001**	0.47 (0.32 – 0.68)	**<0.0001**
> Senior high school	0.26 (0.17 – 0.39)	**<0.0001**	0.27 (0.18 – 0.39)	**<0.0001**	0.27 (0.18 – 0.39)	**<0.0001**	0.27 (0.18 – 0.40)	**<0.0001**	0.27 (0.18 – 0.40)	**<0.0001**
Former smoker	2.59 (1.83 – 3.68)	**<0.0001**	2.58 (1.83 – 3.66)	**<0.0001**	2.58 (1.83 – 3.65)	**<0.0001**	2.63 (1.87 – 3.71)	**<0.0001**	2.59 (1.84 – 3.66)	**<0.0001**
Current smoker	1.11 (0.72 – 1.71)	0.62	1.12 (0.73 – 1.72)	0.59	1.13 (0.74 – 1.72)	0.58	1.13 (0.74 – 1.72)	0.58	1.11 (0.73 – 1.70)	0.62
Diabetes Mellitus	3.51 (2.38 – 5.21)	**<0.0001**	3.67 (2.50 – 5.44)	**<0.0001**	3.72 (2.54 – 5.50)	**<0.0001**	3.71 (2.53 – 5.50)	**<0.0001**	3.76 (2.56 – 5.58)	**<0.0001**
HDL-cholesterol	0.98 (0.97 – 0.99)	**0.003**	0.98 (0.97 – 0.99)	**0.006**	0.98 (0.97 – 0.99)	**0.01**	0.98 (0.97 – 0.99)	**0.01**	0.98 (0.97 – 0.99)	**0.008**
LDL-cholesterol	0.99 (0.98 – 0.99)	**<0.0001**	0.99 (0.98 – 0.99)	**<0.0001**	0.99 (0.98 – 0.99)	**<0.0001**	0.99 (0.98 – 0.99)	**<0.0001**	0.99 (0.98 – 0.99)	**<0.0001**
Systolic Blood Pressure	0.98 (0.97 – 0.99)	**0.05**	0.98 (0.97 – 0.99)	**0.0001**	0.98 (0.97 – 0.99)	**<0.0001**	0.98 (0.97 – 0.99)	**<0.0001**	0.98 (0.97 – 0.99)	**<0.0001**
Antihypertensive medication	12.93 (9.12 – 18.61)	**<0.0001**	13.01 (9.19 – 18.69)	**<0.0001**	13.27 (9.38 – 19.04)	**<0.0001**	13.34 (9.44 – 19.13)	**<0.0001**	13.65 (9.64 – 19.60)	**<0.0001**
Physical activity	0.89 (0.79 – 1.00)	**<0.0001**	0.89 (0.79 – 1.00)	**0.05**	0.89 (0.80 – 1.00)	0.06	0.90 (0.80 – 1.00)	0.06	0.89 (0.80 – 1.00)	0.06
Locality	0.45 (0.12 – 1.59)	0.22	0.62 (0.16 – 2.37)	0.49	0.70 (0.18 – 2.76)	0.61	0.59 (0.15 – 2.37)	0.46	0.88 (0.21 – 3.63)	0.85

All models were adjusted for BMI, age, sex, education, smoking status, diabetes mellitus, HDL-cholesterol, LDL-cholesterol, systolic blood pressure, antihypertensive medication, total physical activity, locality, relative humidity, temperature, and wind velocity. The odds ratio represents the risk for an increase in 1 ppb in ozone or for an increase in 5 of $PM_{2.5}$ μg/m³. Models of

ozone were adjusted for $PM_{2.5}$ in the matching time window and models of $PM_{2.5}$ were adjusted for ozone levels in the matching time window. Note: Since we have 34 localities, we have shown only one OR for locality.
